# Effects of Fish Oil Supplementation on Reducing the Effects of Paternal Obesity and Preventing Fatty Liver in Offspring

**DOI:** 10.3390/nu15245038

**Published:** 2023-12-08

**Authors:** Akriti Shrestha, Sarah Katherine Dellett, Junhui Yang, Upasna Sharma, Latha Ramalingam

**Affiliations:** 1 Department of Nutrition and Food Studies, Syracuse University, Syracuse, NY 13244, USA; akritis4@illinois.edu (A.S.); sdellett@syr.edu (S.K.D.); jyang81@syr.edu (J.Y.); 2 Department of Molecular, Cell and Developmental Biology, University of California Santa Cruz, Santa Cruz, CA 95064, USA; upsharma@ucsc.edu

**Keywords:** epigenetic, intergenerational, omega-3 fatty acids, liver

## Abstract

Nonalcoholic fatty liver disease (NAFLD) is a serious public health concern, which calls for appropriate diet/nutrition intervention. Fish oil (FO) has several benefits in reducing obesity, but its intergenerational role in reducing the effects of paternal obesity has not been established. Hence, we hypothesized that FO supplementation to an obese father during the pre-conceptional period could improve the metabolic health of the offspring, specifically in the liver. Three groups of male mice were fed with a low-fat (LF), high-fat (HF), or high-fat diet supplemented with FO (HF-FO) for 10 weeks and were then allowed to mate with female mice fed a chow diet. Offspring were sacrificed at 16 weeks. The liver tissue was harvested for genomic and histological analyses. The offspring of HF and HF-FO fathers were heavier compared to that of the LF mice during 9–16 weeks. The glucose tolerance of the offspring of HF-FO fathers were significantly improved as compared to the offspring of HF fathers. Paternal FO supplementation significantly lowered inflammation and fatty acid synthesis biomarkers and increased fatty acid oxidation biomarkers in the offspring liver. In summary, FO supplementation in fathers shows the potential to reduce metabolic and cardiovascular diseases through genetic means in offspring.

## 1. Introduction

The rising prevalence of obesity has increased the risk of nonalcoholic fatty liver disease (NAFLD), which currently afflicts approximately 25% of the world’s population [1]. NAFLD is a multi-faceted chronic disease caused by genetic, diet, and environmental factors [2,3,4,5]. It occurs in part through ectopic fat accumulation from the adipose tissue, increased lipolysis and de novo lipogenesis in the liver [6]. NAFLD is highly correlated with obesity as it affects 91% of obese Class II and III patients (BMI > 35 kg/m^2^) [7]. If left untreated, NAFLD could lead to other serious liver diseases: nonalcoholic steatohepatitis (NASH), fibrosis, cirrhosis, and hepatocellular carcinoma [8,9]. The basis of parental obesity’s influence on offspring NAFLD development draws upon the understanding of maternal genetic and gestational factors because these influences have been investigated at length [10,11,12,13,14,15,16]. Research attributes offspring NAFLD development to the genes associated with insulin signaling, lipid homeostasis, and oxidative stress, all known contributors to NAFLD and metabolic disorders [2,17].

Genetic elements from both the mother and father equally contribute to the onset and development of obesity and NAFLD in children, yet the impact of paternal obesity has been scarcely studied [18,19]. Current data show an increasing prevalence of obesity among men of reproductive age [20], which magnifies the need to study the effect of paternal obesity and potential therapies. Researchers have further demonstrated the link between paternal obesity on offspring health via an increased risk of metabolic dysfunction [21], elevated levels of liver alanine aminotransferase (ALT) and serum triglycerides (TG), and lower serum high-density lipoprotein [22]. These elements contribute to hepatic steatosis, i.e., the accumulation of triglycerides in liver [22]. Furthermore, studies have demonstrated that paternal diet and body weight can elicit a metabolic response in offspring: in animal models, a higher sugar intake by fathers during the pre-conceptional period increased the body weight of the offspring [23]. Therefore, this study represents a crucial next step in seeking to attenuate the effects of paternal obesity via supplementation with a known bioactive, fish oil.

At present, the US Food and Drug Administration (FDA) does not recognize any drugs specific for NAFLD treatment [24]. However, ongoing research on therapies for obesity-associated NAFLD includes diet and lifestyle modification for weight loss, the use of the supplements and insulin sensitizers, and surgery [25]. Certain risk factor reduction strategies have been reported in the literature: smoking cessation [26], alternate day fasting [27], consumption of probiotics [28], and supplementation with omega-3 fatty acids (n-3s) [29]. Similarly, improvements in liver histology have been reported: vitamin E, an antioxidant, is shown to lower serum ALT [28], while studies on caffeine intake indicate a decrease in fibrosis, steatohepatitis, and NASH risk [29]. Nevertheless, because NAFLD is associated with obesity, the present focus lies on lowering the risk of obesity-associated NAFLD through a combination of diet modification, weight control, and supplementation with n-3s found in fish oil (FO).

FO contains n-3s docosahexaenoic acid (DHA) and eicosapentaenoic acid (EPA) and is abundant in oily fish, such as trout, salmon, mackerel, and shark. N-3s are demonstrated to reduce the risk of NAFLD through its anti-inflammatory, antithrombotic, and vasodilatory properties [30,31,32]. Research suggests that adult mice fed FO had a reduced fatty liver compared to mice fed sucrose [33]. Furthermore, 16 weeks of FO intervention in obese Sprague–Dawley rats significantly lowered dyslipidemia, hepatic steatosis, and fibrosis compared to rats without FO supplementation [34]. In humans, a pilot study found FO supplementation to be effective in reducing fibrosis in NAFLD patients [35].

The inheritance of FO effects by the offspring has been illustrated in females supplemented with FO starting from 8 weeks before pregnancy through lactation, while their offspring also continued on FO. The offspring continued on FO showed restored glucose tolerance and insulin sensitivity and reduced fatty liver, lipid synthesis, and inflammation [13]. At present, the negative consequences of paternal obesity on offspring health and development are evident; however, an effective intervention for obese fathers during pre-conception is not well studied. Hence, with the many well-established benefits of FO, we aim to investigate the paternal factors and FO’s potential benefits to the metabolic health of the offspring in a mice model.

## 2. Materials and Methods

### 2.1. Animals

Male and female C57BL6J mice aged 4 weeks were purchased from Jackson Laboratory (Bar Harbor, ME, USA) (*n* = 30). After a week of acclimatization, male mice (F_0_) were randomly divided into three dietary groups: a control group with a low-fat diet (LF: 10, 20, and 70% energy from fat, proteins, and carbohydrates, respectively; *n* = 8) and experimental groups with a high-fat diet without (HF: 45, 20, and 35% energy from fat, proteins, and carbohydrates, respectively; n = 11) or with FO supplementation (HF-FO: 36 g/kg of fish oil; *n* = 11). The dietary information is provided in Supplementary Table S1. Fish oil (MEG-3 ultra-high EPA EE oil, catalog no: 5016366) was a kind gift from DSM, Inc (Parsippany, NJ, USA). Female mice were fed a chow diet (Catalog no: 5L0D; Lab diets, Richmond, IN, USA) for the duration of the study. The chow diet contained 14, 28, and 58% energy from fat, proteins, and carbohydrates, respectively. The low-fat and high-fat diets were purchased from Research Diets (New Brunswick, NJ, USA). Room temperature was maintained at 22 °C with 12 h light and dark cycles, and the animals were housed in isolator cages provided with wooden bedding. All animals were provided water and food ad libitum. All animal protocols were approved by the Syracuse University Institutional Animal Care and Use Committee.

### 2.2. Obesity Induction in F_0_ Male Mice and Breeding

Male mice were fed with their respective diets for 10 weeks. Body weights were measured weekly. Weekly food consumption was recorded as the mass difference in food remaining from the food given the previous week. After 10 weeks on the respective diets, each F_0_ male mouse was individually housed with 10-week-old female mouse for conception. Dams continued on a chow diet during gestation (~3 weeks) until weaning.

### 2.3. F_1_ (Offspring Mice) Selection and Body Weight Measurements

The weight of the offspring was recorded on day 1 and day 7. The offspring stayed with their respective female dam until weaning on day 21. Upon weaning, offspring from the three paternal dietary groups were used: LF: *n* = 8 male and *n* = 10 females; HF: *n* = 13 for male and female; and FO: *n* = 13 for male and females. All offspring were fed a chow diet throughout their life. Body weight and food consumption were measured weekly. 

### 2.4. Metabolic Tests

At 12 weeks, a glucose tolerance test was administered to all offspring mice. The baseline blood glucose was measured using a handheld glucometer (Abbott Laboratories, Alameda, CA, USA) following a five-hour fast. After the basal measurement, 2 g/kg glucose was injected intraperitoneally, and subsequently, the blood glucose was measured at 30, 60, 90, and 120 min.

### 2.5. Liver Histology and Triglycerides

The liver tissue of each offspring was fixed in Z fix (Anatech, Ltd., Battlecreek, MI, USA), embedded with paraffin, and stained with hematoxylin and eosin. Tissues were imaged at 20× magnification. Triglycerides were measured in the liver using a triglyceride kit according to the manufacturer’s instructions (Caymen Chemical, Ann Arbor, MI, USA).

### 2.6. Gene Expression

RNA was isolated from the liver using a Zymo Research Quick-RNA MicroPrep Kit (Zymo Research, Irvine, CA, USA). The RNA concentration was measured using Nanodrop (Thermo Fisher, Santa Clara, CA, USA). The RNA was converted to cDNA using a high-capacity kit (Thermo Fisher, Santa Clara, CA, USA). The gene expression of cDNA was conducted using a qPCR with QuantStudio3 PCR machine and its respective primers. The relative gene expression of the target genes was normalized using the HF group as the control and actin as the housekeeping gene, and the relative normalized expression was calculated using the delta delta comparative threshold method (ΔΔCt). The primer sequences are provided in Supplementary Table S2.

### 2.7. Statistical Analyses

To investigate the group differences among the offspring sired by the three dietary groups, a one-way ANOVA was performed using GraphPad Prism, version 9. A two-way ANOVA was performed to determine sex differences and sex–diet interaction.

## 3. Results

### 3.1. F_0_ Male Mice Body Weight

**Figure 1:**
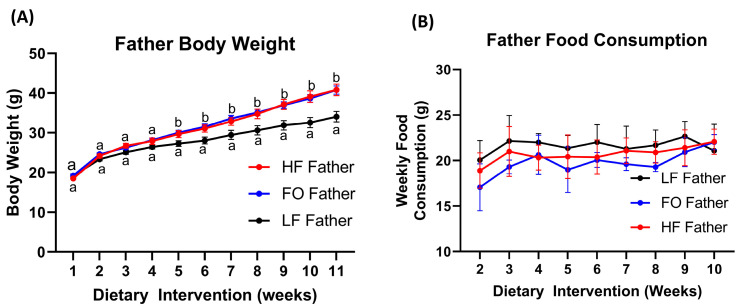
Body weights and food consumption of F_0_ males. The body weight of the mice fathers (F_0_) of the three dietary groups over 10 weeks: LF (*n* = 8), HF (*n* = 11), and FO (*n* = 11) (**A**); weekly food consumption (**B**). Data are presented as the mean ± SEM (*p* < 0.05). Common letters on the error bars indicate no significance (e.g., “a” is significantly different from “b”).

F_0_ fathers randomized to the three dietary groups had comparable body weights (BWs) at the start of the dietary intervention. Furthermore, the BWs between all F_0_ groups were comparable up to 5 weeks of the dietary intervention. The BW of LF fathers was significantly lower compared to those of the HF and HF-FO mice during 5–11 weeks of the dietary intervention (*p* < 0.05), as shown in Figure 1A, though food consumption was similar (Figure 1B). However, no differences in BW were observed between HF and HF-FO fathers throughout the intervention period.

### 3.2. Offspring Body Weight

**Figure 2:**
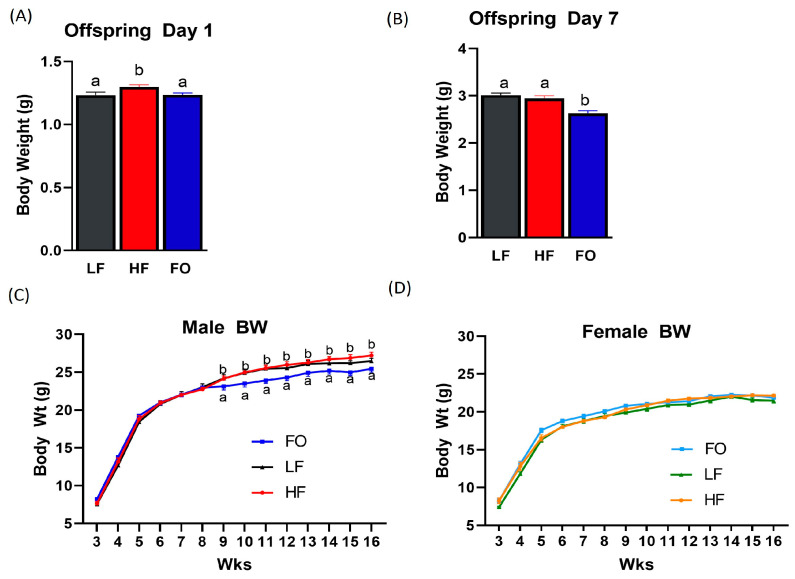
Body weights of the male and female offspring born to fathers fed a LF, HF, and FO diet. The body weight of offspring at birth (**A**). The body weight of offspring at day 7 (**B**). The body weight from weaning at 3 weeks to 16 weeks in males and females, respectively (**C**,**D**). Common letters on the error bars indicate no significance (e.g., “a” is significantly different from “b”).

The offspring mice were weighed on the day of birth (day 1). The offspring born to HF fathers weighed significantly more than those born to LF mice. Furthermore, the offspring born to HF-FO fathers weighed significantly less than the offspring born to HF fathers (Figure 2A). Interestingly, at day 7, the offspring born to HF-FO fathers weighed significantly less than the offspring born to HF and LF fathers (Figure 2B). However, after weaning, male offspring BW was comparable among all groups until 8 weeks of age (Figure 2C). In weeks 9–16, the BWs of the male offspring born to LF fathers and HF fathers were similar, while, interestingly, the offspring born to HF-FO-fed fathers had a significantly reduced BW from 9 weeks to 16 weeks compared to the offspring of both LF- and HF-fed mice fathers. No differences in the BW of the female offspring were observed after the weaning period, as shown in Figure 2D.

### 3.3. Offspring Glucose Tolerance

**Figure 3:**
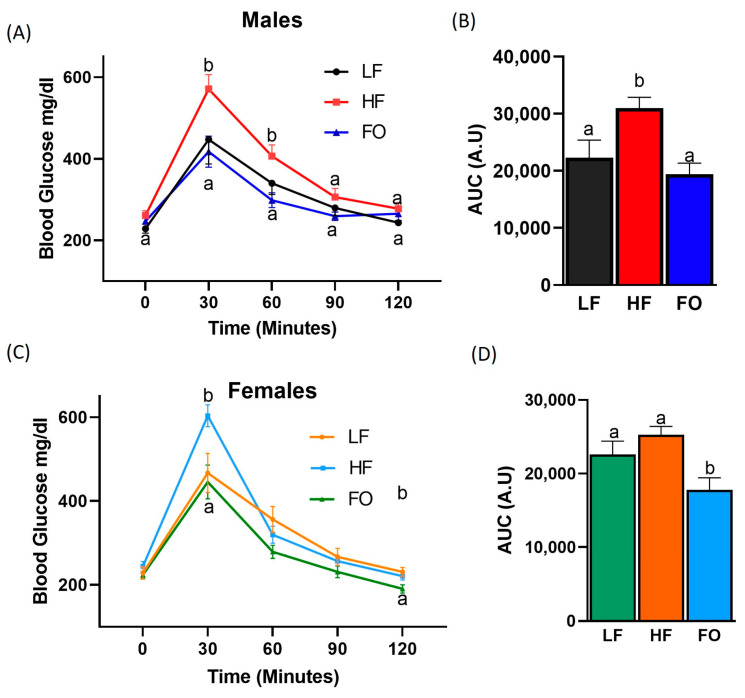
GTT of the male and female offspring. Blood glucose for 2 h after the glucose injection in males (**A**). Area under the curve (AUC) (**B**). Blood glucose for 2 h after the glucose injection in females (**C**), Area under the curve (AUC) (**D**): LF (n = 10), HF (n = 13), and FO (n = 13). Data are presented as the mean ± SEM (*p* < 0.05). Common letters indicate no significance among groups.

No difference was observed in the baseline blood glucose levels in the male offspring sired from the three dietary groups (Figure 3A). After glucose injection, the male HF offspring had significantly higher average blood glucose levels than the LF and HF-FO offspring at 30 and 60 min, suggesting an impaired glucose tolerance. Furthermore, paternal FO supplementation significantly reduced blood glucose levels in the male offspring compared to the offspring of HF-fed fathers at 30 and 60 min. No differences in blood glucose levels were found at 90 and 120 min among groups. Additionally, as shown in Figure 3B, The area under the curve (AUC) for GTT was calculated using the trapezoidal method. The AUC of the HF male offspring was significantly higher than that of the LF offspring, suggestive of insulin resistance. The HF-FO offspring had a significantly lower AUC compared to HF, indicating a better glucose clearance. In the female offspring, blood glucose levels showed no difference at baseline (Figure 3C). As with males, blood glucose level after 30 min of glucose injection was significantly higher in the HF female offspring than in the offspring born to LF fathers. FO attenuated the HF-induced response, as no difference was detected in blood glucose levels between the LF and HF-FO groups at 30 min. No significant differences were observed in blood glucose levels at 60, 90, and 120 min in the female offspring groups. No difference was indicated in the AUC between the LF and HF female offspring. Furthermore, similarly to the males, as indicated by the lower AUC of the FO group, a significantly improved glucose clearance was observed in the female offspring of the HF-FO group compared to the LF and HF groups. These results highlight the effects of paternal FO supplementation in re-establishing the glucose tolerance in the male and female offspring born to obese fathers.

**Figure 4:**
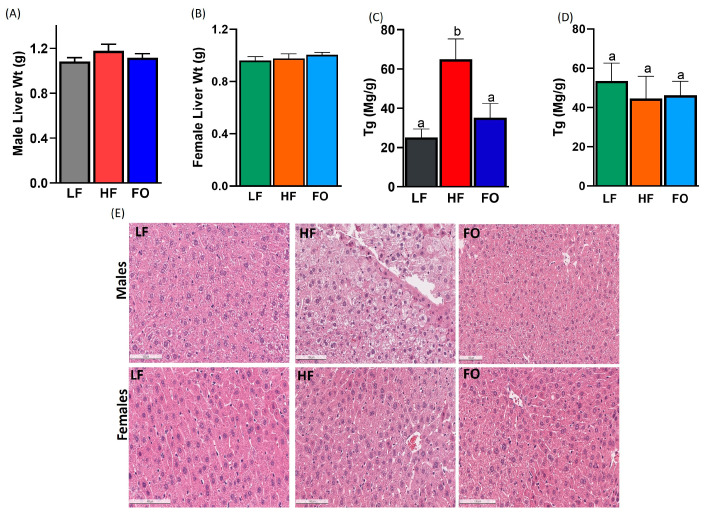
Effects of FO on the liver. Weight of the liver at sacrifice: males (**A**) and females (**B**). Liver triglycerides: males (**C**) and females (**D**). Histological analyses of offspring liver viewed under the microscope (**E**). The images were captured at 20× and scale = 100 µm. Common letters indicate no significance among groups.

The improved glucose clearance with FO could be attributed to potential beneficial effects in the liver. Hence, we focused on the liver mass and function metrics associated with NAFLD. Liver masses were comparable among all dietary groups (Figure 4A,B), suggesting that the liver size alone cannot explain the difference in metabolic function nor does it capture possible inflammatory-induced hepatomegaly. We measured triglycerides in the liver, which were significantly elevated by the HF in the males compared to the LF (Figure 4C). FO significantly lowered triglycerides in the males compared to the HF. No difference in liver triglycerides were observed in the female offspring (Figure 4D). Hematoxylin and eosin staining for the histological comparison of the liver showed that the HF male offspring had more fat deposits than the LF offspring, as indicated by the white fat droplets present. The fat deposits were diminished in the male offspring of FO-supplemented fathers. However, the effect of paternal obesity and FO supplementation was not as apparent in the liver of the female offspring, as shown in Figure 4E, consistent with liver triglycerides. Therefore, we proceeded with the investigation of the liver tissue through gene expression analyses.

### 3.4. Gene Expression Related to Inflammation

**Figure 5:**
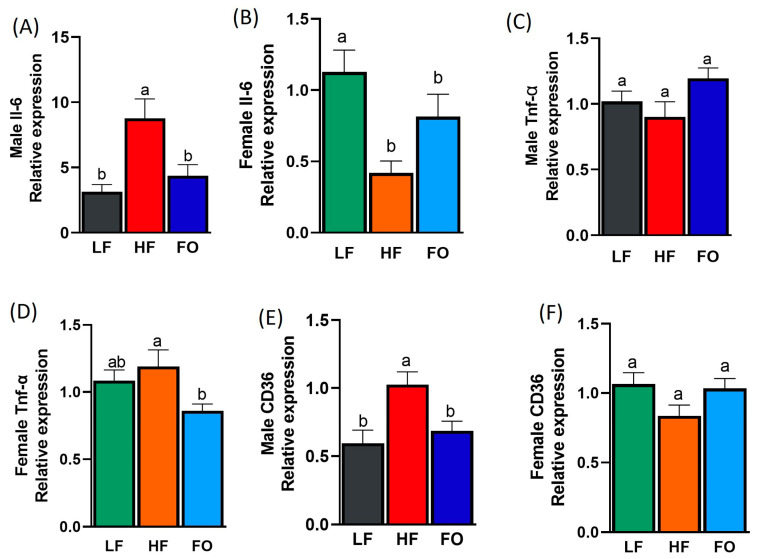
Relative expression of inflammation markers in the livers of the male and female mice. Interleukin-6, (ll6) in males (**A**), Il6 in females (**B**), tumor necrosis factor-alpha, (Tnf-α) in males (**C**) Tnf-a in females (**D**), cluster of differentiation (cd-36) in males (**E**) and cd-36 in females (**F**). Data are presented as the mean ± SEM (n = 8–10, *p* < 0.05). Common letters on the error bars indicate no significance (e.g., “a” is significantly different from “b” and “ab” indicates no significance compared to “a” and “b”).

To better illustrate the alteration of phenotypic tests, genetic markers of lipid metabolism and inflammation markers (interleukin-6 (Il6), tumor necrosis factor alpha (Tnfα), and cluster of differentiation (CD36)) in the liver were measured. Of these markers, we observed pronounced effects by FO to reduce Il6 and CD36 in the male mice. As expected, Il6 levels were significantly higher in the liver of the male offspring from the HF-fed fathers compared to the offspring of the LF-fed fathers (Figure 5A). Furthermore, paternal FO supplementation significantly lowered Il6 levels in its corresponding offspring group compared to the male HF offspring (Figure 5A). However, no differences were observed between the HF and HF-FO female groups for Il6 (Figure 5B). Additionally, statistically significant differences were found regarding Il6 for sex, diet, and sex–diet interaction (Table 1). Females expressed significantly lower levels of Il6 across all three paternal dietary groups relative to their male counterparts. The levels of Tnfα were comparable among both male and female HF and LF offspring groups, as shown in Figure 5C,D. However, paternal FO supplementation only reduced Tnfα in the female offspring but not in males. No sex nor diet differences were observed independently; however, a statistically significant sex–diet interaction was found (Table 1 and Supplementary Table S3). An increase in CD36 levels is associated strongly with liver inflammation and NAFLD [36]. Hence, we measured CD36, which was significantly higher in the male offspring of the HF-fed fathers compared to the offspring of the LF-fed fathers, as shown in Figure 5E. As expected, FO lowered the levels of CD36 in the liver of the corresponding offspring compared to the male offspring of the HF-fed fathers (Figure 5E), with no difference in CD36 expression in females (Figure 5F). The two-way ANOVA showed a statistically significant difference between sexes, though no diet difference was found (Table 1). Additionally, the sex–diet interaction was significant: LF and HF-FO females expressed higher levels of CD36 compared to males. These data clearly illustrate the effect of paternal obesity in increasing the levels of proinflammatory markers, specifically Il6 and CD36, in male offspring as well as the role of paternal FO supplementation in reducing inflammation. 

### 3.5. Gene Expression Related to Fatty Acid Synthesis

**Figure 6:**
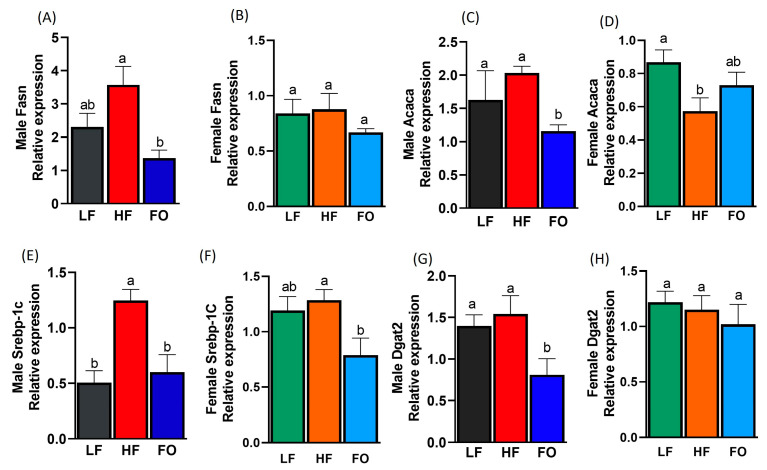
Relative expression of fatty acid synthesis markers in the livers of the male and female mice- Fatty acid synthase (Fasn) in the males and females, respectively (**A**,**B**); acetyl-CoA carboxylase (Acaca) in the males and females, respectively (**C**,**D**); sterol response element binding protein 1c (Srebp-1c) in the males and females, respectively (**E**,**F**); and diacylglycerol O-acyltransferase (Dgat2) in the males and females, respectively (**G**,**H**). Common letters on the error bars indicate no significance (e.g., “a” is significantly different from “b” and “ab” indicates no significance compared to “a” and “b”).

Lipid metabolism is altered with NAFLD. Hence, we analyzed markers for fatty acid synthesis. All markers tested were lowered with FO in males but not in females. Fatty acid synthesis measured using fatty acid synthase (Fasn) was comparable between HF and LF in the male and female offspring (Figure 6A,B). However, FO significantly lowered Fasn levels in the male offspring but not in the female offspring (Figure 6A,B). Furthermore, acetyl carboxylase (Acaca) levels were similar between HF and LF in the male offspring, while HF reduced Acaca levels in the females. Furthermore, FO significantly reduced Acaca levels in the males but not in the females (Figure 6C,D). Statistically significant differences were found between sex, diet, and sex–diet interaction for Acaca and Fasn (Table 1). The female offspring of the LF and HF groups expressed lower levels of Acaca and Fasn compared to the males, though no difference in Fasn and Acaca expression was detected between the HF-FO groups of either sex. We also measured sterol response element-binding protein 1c (Srebp-1c), which was significantly higher in the male offspring of the HF fathers than that of the LF fathers, while FO significantly lowered Srebp-1c compared to the HF group (Figure 6E,F). Similarly, lower levels of Srebp-1c were observed in the female FO group compared to HF, with no difference between the HF and LF groups (Figure 6F). The two-way ANOVA found significant sex, diet, and sex–diet interaction differences for Srepb-1c expression (Table 1). Specifically, the LF males expressed significantly lower Srebp-1c compared to the LF females. We also measured markers for triglyceride accumulation, diacylglycerol O-acyltransferase (Dgat2). The male expression of Dgat2 was similar between the LF and HF groups (Figure 6G), while FO lowered the levels of Dgat2 in males compared to HF (Figure 6G). Comparable levels among all three groups were observed in the females (Figure 6H). A statistically significant difference in Dgat2 expression was found with regard to diet only. No sex difference or sex–diet interaction was indicated.

### 3.6. Gene Expression Related to Fatty Acid Oxidation

**Figure 7:**
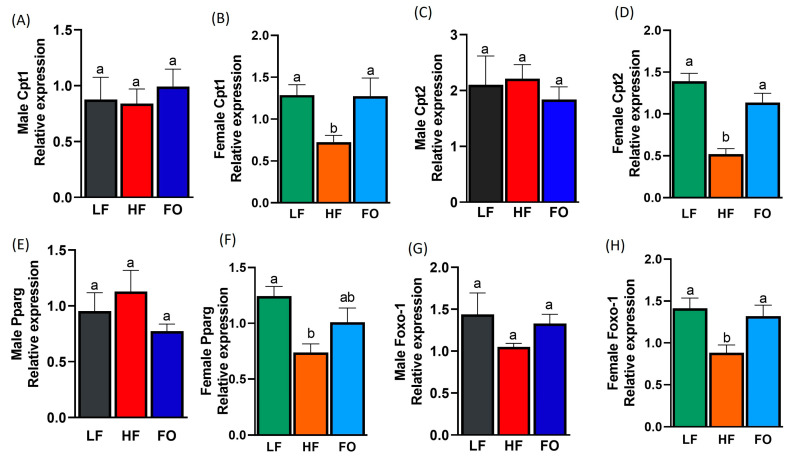
Gene expression of markers related to fatty acid oxidation in the livers of the male and female mice. Relative normalized expression of carnitine palmitoyl-transferase-1 (Cpt1) in the males and females, respectively (**A**,**B**); carnitine palmitoyl-transferase-2 (Cpt2) in the males and females, respectively (**C**,**D**); peroxisome proliferator-activated receptor gamma (Pparg) in the males and females, respectively (**E**,**F**); and forkhead box protein O1 (Foxo-1) in the males and females, respectively (**G**,**H**). Common letters on the error bars indicate no significance (e.g., “a” is significantly different from “b” and “ab” indicates no significance compared to “a” and “b”).

As we observed differences in the fatty acid synthesis, four fatty acid oxidation markers were also measured: carnitine palmitoyltransferase-1 and -2 (Cpt1 and 2), peroxisome proliferator-activated receptor-g (Ppar-g), and forkhead box O1 (Foxo-1). We observed that fatty acid oxidation was increased with paternal FO supplementation in three of the four markers tested, with pronounced effects in the females, but not in the males (Figure 7A–G). The paternal HF diet significantly lowered the levels of both Cpt1 and 2 in the female offspring compared to LF, while FO increased the levels of Cpt1 and 2 compared to HF (Figure 7B,D). No sex or diet differences were found for Cpt1 and 2 with the two-way ANOVA; however, a sex–diet interaction was observed for Cpt2 (Table 1). Specifically, the female offspring of the HF fathers had significantly lower Cpt2 levels than the HF male offspring. HF reduced Ppar-g levels compared to the female offspring born to the LF fathers (Figure 7F). Similar to Cpt2, the two-way ANOVA found a sex–diet interaction for Pparg, with lower Pparg levels in the females of the HF-fed fathers than the males (Table 1). Lastly, HF females expressed lower levels of Foxo-1 compared to the LF group, while Foxo-1 expression was significantly higher in the HF-FO females compared to the HF group (Figure 7H). Only diet differences were found for Foxo-1 (Table 1).

### 3.7. Gene Expression Related to Carbohydrate Metabolism

**Figure 8:**
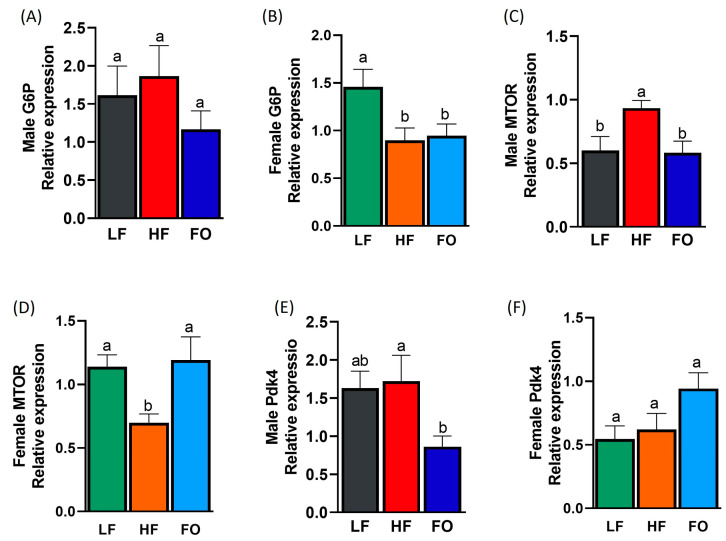
Gene expression of the markers related to carbohydrate metabolism in the livers of the male and female mice. Relative normalized expression of glucose-6-phosphatase (G6P) in the males and females, respectively (**A**,**B**); the mammalian target of rapamycin (mTOR) in the males and females, respectively (**C**,**D**); and pyruvate dehydrogenase kinase 4 (Pdk4) in the males and females, respectively (**E**,**F**). Common letters on the error bars indicate no significance (e.g., “a” is significantly different from “b” and “ab” indicates no significance compared to “a” and “b”).

The genes governing carbohydrate metabolism were also evaluated: glucose-6-phosphatase (G6Pase), the mammalian target of rapamycin (mTOR), and pyruvate dehydrogenase kinase 4 (Pdk4). The males had similar levels of G6Pase (Figure 8A). In the females, paternal HF supplementation lowered the levels of G6Pase compared to the LF group, with no differences in G6Pase expression between the HF and HF-FO female offspring (Figure 8B). Only difference in sexes was found for G6Pase expression (Table 1). Specifically, the levels of G6Pase in the HF females were significantly lower than those in the HF males. mTOR expression was elevated in the male offspring from the HF-fed fathers compared to the LF group but was attenuated in comparison to the FO group (Figure 8C). In the females, the paternal HF diet lowered mTOR levels, while paternal FO supplementation significantly increased it (Figure 8D). A statistically significant difference in mTOR due to sex and sex–diet interaction was found, with no difference for diet. (Table 1). The female offspring of the LF and FO supplementation groups had higher levels of mTOR than the corresponding male groups. Lastly, no difference in Pdk4 expression was observed between the male and female offspring of the HF and LF fathers (Figure 8E,F). Paternal FO supplementation significantly lowered the levels of Pdk4 in the male offspring, while Pdk4 expression was similar among all female offspring groups. Like mTOR, a significant difference in Pdk4 expression due to sex and sex–diet interaction, but not diet exclusively, was found (Table 1). The female offspring of the LF and HF fathers had lower Pdk4 levels compared to the males of the LF and HF fathers, respectively, though no difference was indicated between the offspring groups of FO parentage.

## 4. Discussion

Paternal obesity has adverse effects on offspring health and development, specifically through intergenerational programming [37]. The pre-conceptional risk factors of fathers, including but not limited to obesity, diabetes mellitus, nutritional or dietary habits, obesogenic lifestyles, and substance abuse, are known to modulate offspring phenotypes and are likely mediated through the impaired molecular profile of spermatozoa and alteration in sperm epigenome [38]. Consistent with these findings, our results show that the offspring of the HF-fed fathers had a higher birth weight at day 1 compared to the offspring of the LF-fed fathers, illustrating an effect of paternal obesity on offspring body weight at the earliest stages of life. This result is independent of the effects of maternal obesity as dams were fed a chow diet during paternal dietary intervention period and throughout mating, pregnancy, and weaning. Similar to our study, other research has shown a link between paternal diet and offspring body weight: the higher body weight of offspring was observed due to a higher sugar consumption by the father in *Drosophila* study [39] or HF-diet-induced paternal obesity in mice [37,40,41]. Nevertheless, studies providing effective interventions to reduce the effect of paternal obesity on offspring are scarce and usually focus only on intervention through physical exercise rather than dietary supplementation [40].

The present work provides evidence of FO supplementation in mitigating childhood obesity as well as reducing the programming effect of paternal obesity on offspring health and metabolic parameters using animal models. Our study showed that, although FO fathers had a similar body weight as that of HF fathers, the body weight was reduced in the male offspring of fathers supplemented with FO. Specifically, the male offspring of the HF-FO fathers had a significantly lower body weight than those of obese fathers fed a HF diet during weeks 9–16 of life. Moreover, FO restored glucose tolerance in offspring to levels similar to those born to fathers on a LF diet, indicating that FO has a metabolic effect outside of weight control alone. Interestingly, no difference in the body weight of the female offspring was observed. This could be attributed to the estrogen levels in the females or the strain of mice (C57BL6) employed in this study, which exhibit a more favorable short-term response to weight gain with a HF diet in males when compared to females [42]. However, in the absence of body weight differences in the female offspring, paternal FO supplementation demonstrated beneficial effects on glucose tolerance. 

To comprehend the development of fatty liver leading to NAFLD, liver histology was analyzed, which revealed a higher fat deposition in the offspring livers of the HF-fed fathers compared to the offspring livers of the LF-fed fathers. Furthermore, the defining characteristic of NAFLD is the abnormal buildup of triglycerides (TG) in the liver [43]. Capel et al. showed that Srebp-1c, a transcription factor for lipogenic genes, is upregulated with chronic HF feeding [44]. In line with this, we found a significantly higher expression of fatty acid synthesis markers Srebp-1c and Fasn in the livers of the male offspring of the HF-fed fathers compared to the male offspring of the LF-fed fathers. N-3s are known to reduce TG accumulation via genetic mechanisms by reducing levels of lipogenic genes [45]. Accordingly, with FO, we observed lower levels of Srebp-1c and Fasn, as well as Acaca, in the male offspring, suggesting the benefit of FO supplementation during paternal obesity. Furthermore, both the male and female offspring of FO-supplemented fathers had lower levels of Dgat2, an enzyme responsible for TG synthesis. Furthermore, the activation of mTOR plays a crucial role in the regulation of lipogenesis by facilitating Srebp-1c [46]. We additionally saw a decrease in mTOR levels in the male offspring with FO, but not in the females, suggesting FO reduces lipogenesis by multiple and varying pathways in both sexes. This is particularly significant for insulin resistance etiologies originating from liver, as insulin-mediated pathways, including the cellular uptake of glucose, fatty acid synthesis, and fatty acid oxidation, are disrupted with obesity and liver pathologies [47].

It is well established that FO enhances fatty acid oxidation in the liver [48]. Interestingly, in our study, we observed FO to improve fatty acid oxidation in the females but not in the males, indicating sex differences govern lipid metabolism. The higher fatty oxidation levels observed among females could be contributed in part to the higher adiponectin levels of females compared to males [49]. Furthermore, female C57BL6J mice are known to be more insulin-sensitive, and therefore less susceptible to the development of insulin resistance, than male C57BL6J mice, which in turn improves the rates of fatty acid oxidation [42]. 

A long-term HF diet is known to cause increased adipocyte size with excess fatty acid production. These excess fatty acids from adipocytes are transported to the liver along with inflammatory adipokines, conditions that lead to NAFLD [50]. Our findings confirm that there is a higher expression of pro-inflammatory markers, including Il6, Tnfα, and CD36, in the liver of the offspring of the HF fathers as compared to the offspring of the LF fathers. Such programming of fathers’ obesity in the offspring is shown to impair the adipokine levels in the offspring up to the next two generations [11,12,18]. Furthermore, FO is an established anti-inflammatory agent [48], and our study showed FO to lower inflammation in the liver via intergenerational mechanisms. We found a lower expression of Il6, Tnfα, and CD36 in the male offspring of the FO fathers compared to the offspring of the HF fathers, while only Tnfα was reduced in the female offspring, indicating sex-dependent effects. A notable variation in Tnfα expression between sexes was recorded. This suggests that distinct pathways are modified in males and females in reaction to FO. This phenomenon might be attributed to the metabolites of EPA and DHA, referred to as specialized pro-resolving mediators (SPMs), which are more abundant in female mice in comparison to their male counterparts [51]. This study and others found that the administration of these secondary metabolites of n-3 PUFAs lower inflammatory response. Furthermore, females are less prone to inflammatory response in part due to the naturally higher levels of SPMs caused by sex-specific metabolic differences. 

Because of the personal health benefits of n-3s outlined in this paper and elsewhere in the literature, the current general public recommendation by the WHO is the intake of at least 2–3 oz portions of fatty fish, providing at a minimum 1–3 g FO, per week, while the Global Organization for EPA and DHA Omega-3s recommends a daily combined intake of 500 mg EPA and DHA [52]. For cardiovascular disease (CVD) patients, some fish oil products are approved by the FDA as prescription medications at doses up to 4 g per day, since they are effective in reducing TG in adults by 20–50% [53]. In our study, we used 36 g FO per kg of mouse diet, which approximates to 11 g FO per day in humans. This amount surpasses the present guideline of 2–4 g FO/day for those with high serum TG. Nevertheless, research involving humans has been conducted using doses as high as 12 g/day [54]. 

## 5. Conclusions

For the first time, the findings of the current study show that supplementing FO in the father’s diet improved the offspring metabolic health. It is intriguing that these effects were observed with the alteration of the father’s diet only, with no alteration to the mother or the offspring diets in this study. Additionally, these metabolic improvements via dietary supplementation were observed despite there being no differences in body weights among fathers. Hence, future work may provide additional insight into obesity prevention with the combination of weight loss and FO supplementation for the fathers, given the positive association between weight control, lower inflammation, and cardiovascular risk factors. Pre-conceptional risk factors in the father are mediated through sperm RNAs for programming offspring phenotypes [19], which needs to be addressed in the future. Additionally, further studies should explore the vast sex differences in genetic programming due to paternal intergenerational influence.

## Figures and Tables

**Table 1:** ANOVA—diet and sex differences across the groups.

	Sex (S)	Diet (D)	Interactions (S × D)
Il-6	<0.05	<0.05	<0.05
Tnf-α	0.90	0.94	<0.05
CD36	<0.05	0.48	<0.05
Fasn	<0.05	<0.05	<0.05
Acaca	<0.05	<0.05	<0.05
Srepb-1c	<0.05	<0.05	<0.05
Dgat2	0.40	<0.05	0.20
Cpt-1	0.14	0.07	0.23
Cpt-2	0.06	0.28	<0.05
Ppar-g	0.72	0.20	<0.05
Foxo-1	0.57	<0.05	0.84
G6Pase	<0.05	0.21	0.26
mTOR	<0.05	0.97	<0.05
Pdk4	<0.05	0.30	<0.05

Differences in sex and diet and whether an interaction exists between the groups were calculated.

## Data Availability

Data are contained within the article.

## Supplementary Materials

The following supporting information can be downloaded at: https://www.mdpi.com/article/10.3390/nu15245038/s1, Supplemental Table S1: Diet Composition. Supplemental Table S2. Supplemental Table S3: F value for ANOVA analyses (F (DFn, DFd).
