# Supplementary material for: Effects of Fish Oil Supplementation on Reducing the Effects of Paternal Obesity and Preventing Fatty Liver in Offspring
Source: Nutrients. 2023 Dec 8;15(24):5038. doi: 10.3390/nu15245038 (PMC10745816; doi:10.3390/nu15245038)
Supplement: Supplementary file 1 [file nutrients-15-05038-s001.zip › nutrients-2695250-supplementary.pdf]

Supplemental Table 1: Diet Composition

	**Control- Low Fat**		**High Fat**		**High Fat – Fish Oil**	
	gm%	*kcal%*	gm%	*kcal%*	gm%	*kcal%*
Protein	28	*28.5*	34	*28.5*	34	*28.5*
Carbohydrate	57	*58.0*	31	*27*	31	*27*
Fat	6	*13.5*	24	*45*	24	*45*
Total		*100*		*100*		*100*
kcal/gm	3.92		4.7		4.7	
**Ingredient**	**gm**	***kcal***	**gm**	***kcal***	**gm**	***kcal***
Casein, 30 mesh	286.1	*1144*	286	*1144*	286	*1144*
L-Cystine	3	*12*	3	*12*	3	*12*
Corn Starch	330	*1320*	11.8	*47*	11.8	*47*
Maltodextrin 10	75	*300*	75	*300*	75	*300*
Sucrose	172.8	*691*	172.8	*691*	172.8	*691*
Cellulose	50	*0*	50	*0*	50	*0*
Soybean Oil	25	*225*	25	*225*	25	*225*
Lard	36	*324*	177.5	*1598*	134.6	*1211*
MEG-3 Ultra High EPA EE oil (720 mg/g EPA)	0	*0*	0	*0*	42.91	*386*
Mineral Mix, S10026	10	*0*	10	*0*	10	*0*
DiCalcium Phosphate	13	*0*	13	*0*	13	*0*
Calcium Carbonate	5.5	*0*	5.5	*0*	5.5	*0*

Potassium Citrate, 1 H2O		16.5	*0*		16.5	*0*		16.5	*0*
Vitamin Mix, V10001		10	*40*		10	*40*		10	*40*
Choline Bitartrate		2	*0*		2	*0*		2	*0*
Vitamin E Acetate, 50% (500 IU/gm)		0	*0*		0.13	*0*		0.13	*0*
FD&C Yellow Dye #5		0	*0*		0	*0*		0.05	*0*
FD&C Red Dye #40		0.05	*0*		0.025	*0*		0	*0*
FD&C Blue Dye #1		0	*0*		0.025	*0*		0	*0*
**Total**		**1034.95**	***4057***		**858.28**	***4057***		**858.29**	***4057***

Supplemental Table -2

Gene	Forward	Reverse
Acaca	GCAGCAGTTACACCACATAC	TCCGCCATCTTCCACAATA
Actin	TGAGACCTTCAACACCCCAGCCA	CGTAGATGGGCACAGTGTGGGTG
Cd36	AGGTCTATCTACGCTGTGTTC	AGGCATTGGCTGGAAGAA
Cpt-1	AAGGAATGCAGGTCCACATC	CCAGGCTACAGTGGGACATT
Cpt-2	GACCAGTGAGAACCGAGAT	AAGCAGAGGCAGAAGACA
Dgat2	TCCAGAAGAAGTTCCAGAAGTAT	CAGGTGTCAGAGGAGAAGAG
Fasn	GTCGTCTATACCACTGCTTACT	ACACCACCTGAACCTGAG
Foxo1	GCTCTGTCCTGAAGAATCCT	CTAATCCTGCCACTGTCTGTA
G6Pase	AACGCCTTCTATGTCCTCTTTC	GTTGCTGTAGTAGTCGGTGTCC
IL-6	TCAGGTCAGCAATCACAGA	ACAGGTCTGTTGGGAGTGGT
mTOR	CAGCAACAGTGAGAGTGAAG	ACAAGGAGATAGAACGGAAGAA
Pdk4	TGTGATGTGGTAGCAGTAGT	TGGTGAAGGTGTGAAGGA
Ppar-g	ACGATCTGCCTGAGGTCTGT	CATCGAGGACATCCAAGACA
Srepb-1c	TCTGCCTTGATGAAGTGTGG	AGCAGCCCCTAGAACAAACA
Tnf-α	TCTCAAAACT CGAGTGACAAGC	GGTTGTCTTTGAGA TCCATGC

Supplemental Table 3: F value for ANOVA analyses (F (DFn, DFd)

	**Diet (D)**	**Sex (S)**	**Interactions (S x D)**
Il-6	F (2, 43) = 9.327	F (1, 43) = 86.92	F (2, 43) = 13.19
Tnf-α	F (2, 48) = 0.06081	F (1, 48) = 0.01553	F (2, 48) = 5.211
Cd36	F (2, 43) = 0.7460	F (1, 43) = 9.770	F (2, 43) = 8.581
Fasn	F (2, 37) = 6.446	F (1, 37) = 32.45	F (2, 37) = 4.537
Acaca	F (2, 45) = 3.261	F (1, 45) = 49.90	F (2, 45) = 6.259
Srepb-1c	F (2, 43) = 9.825	F (1, 43) = 7.552	F (2, 43) = 3.264
Dgat2	F (2, 39) = 4.253	F (1, 39) = 0.7363	F (2, 39) = 1.680
Cpt-1	F (2, 41) = 2.884	F (1, 41) = 2.301	F (2, 41) = 1.498
Cpt-2	F (2, 48) = 1.296	F (1, 48) = 29.37	F (2, 48) = 3.046
Ppar-g	F (2, 41) = 1.646	F (1, 41) = 0.1245	F (2, 41) = 5.690
Foxo-1	F (2, 43) = 5.488	F (1, 43) = 0.3234	F (2, 43) = 0.1721
G6Pase	F (2, 49) = 1.607	F (1, 49) = 4.042	F (2, 49) = 1.383
mTOR	F (2, 50) = 0.02635	F (1, 50) = 14.00	F (2, 50) = 7.370
Pdk4	F (2, 33) = 1.249	F (1, 33) = 23.98	F (2, 33) = 7.471
